# Reactive oxidative species (ROS)-based nanomedicine for BBB crossing and glioma treatment: current status and future directions

**DOI:** 10.3389/fimmu.2023.1241791

**Published:** 2023-09-04

**Authors:** Dandan Wu, Xuehui Chen, Shuqiu Zhou, Bin Li

**Affiliations:** ^1^Department of Radiology, The First People’s Hospital of Linping District, Hangzhou, China; ^2^Department of Radiology, Tongjiang People’s Hospital, Tongjiang, China; ^3^Department of Geriatrics, The Fourth Hospital of Daqing, Daqing, China

**Keywords:** reactive oxygen species (ROS), nanomedicine, glioma, blood-brain barrier (BBB), photodynamic therapy

## Abstract

Glioma is the most common primary intracranial tumor in adults with poor prognosis. Current clinical treatment for glioma includes surgical resection along with chemoradiotherapy. However, the therapeutic efficacy is still unsatisfactory. The invasive nature of the glioma makes it impossible to completely resect it. The presence of blood-brain barrier (BBB) blocks chemotherapeutic drugs access to brain parenchyma for glioma treatment. Besides, tumor heterogeneity and hypoxic tumor microenvironment remarkably limit the efficacy of radiotherapy. With rapid advances of nanotechnology, the emergence of a new treatment approach, namely, reactive oxygen species (ROS)-based nanotherapy, provides an effective approach for eliminating glioma via generating large amounts of ROS in glioma cells. In addition, the emerging nanotechnology also provides BBB-crossing strategies, which allows effective ROS-based nanotherapy of glioma. In this review, we summarized ROS-based nanomedicine and their application in glioma treatment, including photodynamic therapy (PDT), photothermal therapy (PTT), chemodynamic therapy (CDT), sonodynamic therapy (SDT), radiation therapy, *etc.* Moreover, the current challenges and future prospects of ROS-based nanomedicine are also elucidated with the intention to accelerate its clinical translation.

## 1 Introduction

Glioma is the most common primary intracranial tumor in adults, with an annual incidence of 3-6.4 per 100,000, accounting for 23.3% of all central nervous system (CNS) tumors and 78.3% of CNS malignant tumors (1). Among them, the WHO grade IV glioblastoma has the highest annual incidence rate (4.03 per 100,000), accounting for 48.6% of all primary CNS malignancies (1). Recent studies have shown that glioblastoma has surpassed liver cancer and pancreatic cancer to become the first refractory tumor (2). Current clinical treatment for glioma includes maximally surgical resection, radiotherapy and chemotherapy. However, most gliomas deeply infiltrate into the surrounding normal cerebral tissue, making it impossible to resect completely (3). With unlimited tissue penetration depth, radiotherapy can damage DNA and kill tumor cells, but its efficiency is often limited by several factors, such as hypoxic tumor microenvironment, low radiation absorption of tumor tissues (4). Chemotherapy, as the most commonly used anti-cancer approach, has a limited therapeutic effect on glioma. The existence of the blood-brain barrier (BBB) allows only 1-2% of the chemotherapeutic drugs enter the glioma sites, and the rest of the chemotherapeutic drugs are distributed in other normal tissues and organs, inducing serious toxic and side effects (5). Additionally, the high heterogeneity, hypoxia, and presence of tumor stem cells in glioma cause drug resistance in tumor tissue (6, 7). Therefore, it is urgent to explore and develop new treatment approach for glioma.

The BBB was first proposed by Edwin Goldman in 1913, but the definite structure was only known to scientists when the scanning electron microscope appeared in 1937 (8). Specifically, BBB is a highly selective semipermeable barrier between blood plasma and neural cells, which can selectively prevent certain substances in blood circulation from entering the brain (5). The BBB is important for the homeostasis of the CNS. The abnormal disruption of BBB might lead to nervous dysfunction. The BBB mainly contains brain capillary endothelial cells (BCECs), end-feet of astrocytes, and pericytes (9). Among them, there are tight junctions between BCECs, which can strictly control the diffusion of small water-soluble molecules through the paracellular pathway. As a biochemical and physical barrier, the intact BBB can effectively restrict more than 98% of small molecule drugs and almost all macromolecular drugs from entering the CNS (10). Small fat-soluble molecules smaller than 400–500 Da (such as oxygen, CO_2_, steroid hormones) can freely enter and exit via transcellular pathways. Some essential nutrients (such as glucose, electrolytes, *etc.*) can enter the brain via carrier-mediated transcytosis (CMT) pathway. For example, the glucose transport receptor 1 (GLUT1) can facilitate the transport of glucose (11). Substances such as transferrin (Tf) need to enter the brain through the receptor-mediated transcytosis (RMT) pathway, and they can be absorbed by specific receptors on the BBB (such as low-density lipoprotein receptor, transferrin receptor). Cationic proteins can electrostatically adsorb to anionic sites on the surface of BCEC membranes and enter the brain through adsorption-mediated transcytosis (AMT). In addition, current studies have found that immune cells and stem cells can span the BBB through cell-mediated transport pathways (12, 13). Previous studies have shown that the BBB in glioma could be partly disrupted during rapid tumor growth (14, 15). However, the BBB in glioma remains a hindrance for most chemotherapeutic drugs. The presence of BBB can limit the entry of chemotherapeutic drugs into the CNS, thereby restricting effective treatment of glioma. Notably, recent development of nanotechnology have produced a large number of nanoparticles capable of crossing or bypassing the BBB, enabling effective theranostics of glioma (5). For example, surface modifications of BBB/glioma-targeting peptides or cell-penetration peptides allow the nanoparticles to effectively span the BBB and further precisely target tumor cells (16–19). Besides, GLUT1, the most common transporter in CMT pathway, which is widely expressed on BCECs, and its expression level is significantly higher than that of other transporters. Anraku et al. constructed a glucosylated nanocarrier and promoted the its efficiency of crossing BBB by regulating blood glucose level (20, 21). In addition, the several cell types or their nanovesicles, such as neural stem cells, mesenchymal stem cells, and macrophages, *etc*, could effectively cross the BBB (22–25). Furthermore, the microbubble-mediated focused ultrasound could also open the BBB with the acoustic cavitation effect (26–28).

## 2 Reactive oxygen species-based nanomedicine

Generally, reactive oxygen species (ROS) is a series of chemicals with a high degree of oxidative activity, including hydrogen peroxide (H_2_O_2_), singlet oxygen (^1^O_2_), superoxide anion (O_2_^•−^), and hydroxyl radical (•OH) (29). The overexpressed ROS in tumor cells could induce imbalance of oxidative stress, resulting in severe damage to tumor cells. The tumor cells could generate ROS at various subcellular locations. For instance, the electrons escaped from respiratory chain in mitochondria can convert oxygen into O_2_^•−^, which can further react to produce other ROS (*e.g.*, H_2_O_2_, •OH, *etc.*) through different reactions (30). High reactivity is the major feature of the ROS, resulting in various ROS-mediated reaction in tumor cells, including reduction, oxidation, and dismutation. According to the reaction principles, the ROS is divided into two types: one-electron oxidants (*e.g.*, O_2_^•−^, •OH, *etc.*) and two-electron oxidants (H_2_O_2_) (31). In addition, compared with normal cells, the ROS content is significantly increased in tumor cells, which was associated with the tumorigenesis of malignancies (32, 33). However, the excessive level of ROS can result in oxidative injury of cells. With intracellular antioxidant defense mechanisms, the antioxidants, such as glutathione (GSH), are also highly expressed in tumor cells, which keeps the redox homeostasis in tumor cells (34, 35). In recent years, with the rapid development of nanotechnology, the ROS-based nanomedicine has been widely applied in cancer treatment, including photodynamic therapy (PDT), photothermal therapy (PTT), chemodynamic therapy (CDT), sonodynamic therapy (SDT), radiation therapy, *etc* (36–38). These treatment approaches utilize nanosensitizers and exo/endogenous stimulus (*e.g.*, light, ultrasound, X-ray, H_2_O_2_, *etc.*) to generate ROS in tumor cells, thereby disrupting the redox balance and effectively kill the tumor cells.

## 3 Application of ROS-based nanomedicine in glioma treatment

### 3.1 Photodynamic therapy (PDT)

PDT, the first ROS-based treatment for cancer regression, was mentioned in Raab’s introduction of light-related cytotoxicity in 1990. There are three key components for PDT: light, photosensitizer (PS), and intratumoral oxygen. The laser could activate the PSs to convert their excited-state energy to surrounding oxygen to produce ROS, which augments the oxidative stress in cancer cells and further kill the cancer cells (39). Since the PSs could only induce ROS generation upon light irradiation, the PDT is highly selective in eradicating tumors with little effect on healthy organs (*e.g.*, heart, spleen, liver, kidney, *etc.*). In addition, PDT could be divided into two main subtypes according to photochemical reaction mechanisms: type I PDT and type II PDT. Upon activation of laser, the PSs convert the ground singlet state to the excited triple state and then trigger the photochemical reaction through two pathways (type I and type II). For type I PDT, the PSs catalyze biological substrate to produce radical intermediates which further interact with water and triplet oxygen (^3^O_2_) to produce hydroxyl radicals (·OH) and superoxide anions (O_2_^·-^). PSs transfer directly the energy to the surrounding ^3^O_2_ into singlet oxygen (^1^O_2_) for the type II PDT. Thus, type I PDT is oxygen-independent, while the type II PDT can only occur in well-oxygenated microenvironment.

Nowadays, PDT has been widely used in cancer treatments in clinics, including lung cancer, esophageal cancer, skin cancer, and glioma, *etc.* The 5-aminolevulinic acid (5-ALA), an intermediate metabolite in hemoglobin pathway, has been approved by United States Food and Drug Administration (FDA) for fluorescence imaging-guided surgical resection of solid tumors. In recent years, the 5-ALA has been gradually utilized as a PS for PDT of glioma. 5-ALA could accumulate in glioma cells after oral administration, which is transformed into protoporphyrin (PpIX) in the mitochondria. Upon light irradiation, the PpIX could induce large amount of ROS in glioma cells, which could trigger imbalance of oxidative stress and cause cell death. However, there are several challenges for the 5-ALA-based PDT, including lacking of active targeting ability, short wavelength of excitation wavelength, insufficient tissue penetration depth, and inefficient in hypoxic environments, *etc.*

**Figure 1:**
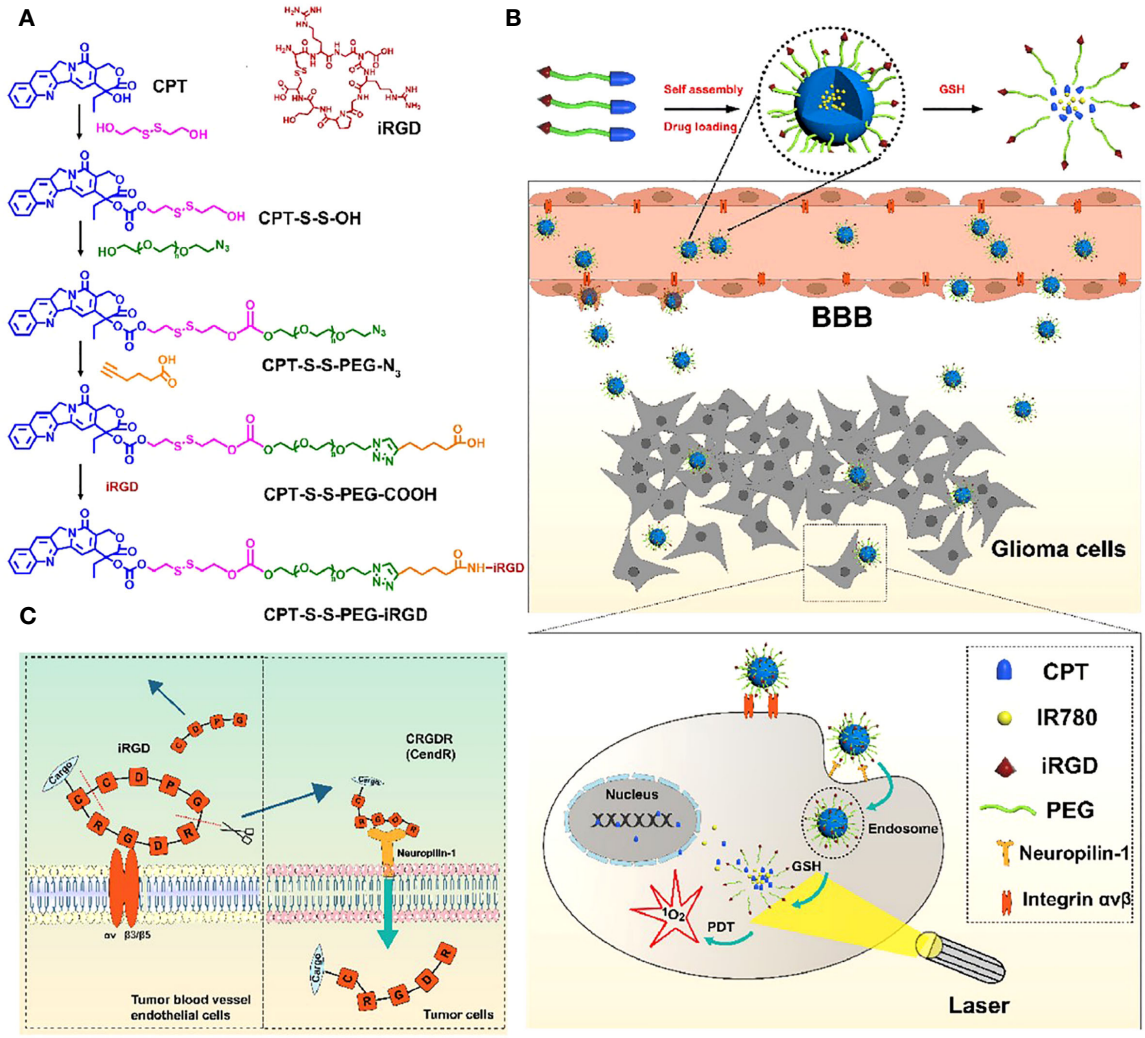
**(A)** Chemical structure of the prepared CPT-S-S-PEG-iRGD nanopolymer. **(B)** Schematic illustration of BBB penetration and tumor cell targeting of the prepared polymers. **(C)** The mechanism of iRGD for BBB penetration and tumor cell targeting. Reproduced with permission from ref (40). Copyright 2020, Elsevier.

With rapid development of nanotechnology, the nanomedicine-mediated PDT has widely applied in treatment of glioma (**Table 1**) (42, 43, 52–58). For example, Cai group developed a disulfide bond-conjugated polymer for targeted PDT and chemotherapy of orthotopic glioma (40). The camptothecin (CPT)-S-S-PEG-COOH was able to assemble into polymeric micelles which were further loaded with IR780 as PS and modified with iRGD peptide for BBB penetration and tumor targeting (**Figures 1A, B**). Due to the conjugation of iRGD peptide, the CPT-S-S-PEG-iRGD@IR780 micelles could effectively span the BBB and actively target tumor cells through interaction between iRGD peptide and αvβ integrin (**Figure 1C**). The *in vivo* experiments showed that these micelles could effectively deliver the IR780 into the orthotopic glioma region, leading to excellent anti-tumor effect upon laser irradiation. Overall, the CPT-S-S-PEG-iRGD@IR780 micelles were an ideal delivery nano carrier of PS (IR780) and chemotherapeutic drug (CPT), and the micelle-mediated PDT is a promising treatment for glioma. In addition, to enhance the tumor accumulation of PSs, Chen group proposed a novel drug delivery strategy in which the platelet was used as carrier of PSs (41). They loaded the chlorine e6 (Ce6) into boron nitride nanoparticles and further coated with doxorubicin and polyglycerol (BNPD-Ce6). Then, the BNPD-Ce6 nanoparticles were loaded into mouse platelets to form the BNPD-Ce6@Plt nanocarriers. The *in vivo* experiment showed that the BNPD-Ce6@Plt could rapidly accumulated in both subcutaneous and orthotopic GL261 tumors after intravenous administration. Upon light irradiation at a wavelength of 808 nm, the BNPD-Ce6@Plt nanoparticles could produce amount of ROS, inducing DNA damage and tumor cell death. *In vivo* therapeutic efficacy evaluation revealed that the BNPD-Ce6@Plt-mediated PDT remarkably regress the orthotopic glioma and prolong the survival time of mouse.

**Table 1:** ROS-based nanomedicine for glioma treatment.

Nanomedicine	ROS treatment	Sensitizers	ROS types	Mouse model	Synergistic therapy	Ref
CPT-S-S-PEG-iRGD@IR780	PDT	IR780	^1^O_2_	U87 orthotopic glioma-bearing mice	Na	(40)
BNPD-Ce6@Plt	PDT	Ce6	^1^O_2_	GL261-luc orthotopic glioma-bearing mice	Na	(41)
Fe_3_O_4_@mSiO_2_(DOX)@HSA(Ce6)	PDT	Ce6	^1^O_2_	U87 glioma-bearing mice	Chemotherapy	(42)
ptHDL/siHIF-ICG	PDT	ICG	^1^O_2_	U87 orthotopic glioma-bearing mice	Gene therapy	(43)
CuFeSe2-LOD@Lipo-CM	CDT	Cu^+^	•OH	U87 orthotopic glioma-bearing mice	Photothermal therapy	(44)
UMDL nanodrug	CDT, PDT	NH2-MIL-53 (Fe)	•OH, ^1^O_2_	GL261 orthotopic glioma-bearing mice	Chemotherapy	(45)
AMGDC nanocatalyst	CDT	Mn^2+^	•OH	U87 glioma-bearing mice	Photothermal therapy	(46)
MnO_2_@Tf-ppIX	SDT	ppIX	^1^O_2_	C6 orthotopic glioma-bearing mice	na	(47)
ACHL	SDT	Ce6	^1^O_2_	GL261 orthotopic glioma-bearing mice	autophagy inhibition	(48)
CSI@Ex-A	SDT	ICG	^1^O_2_	U87 orthotopic glioma-bearing mice	Na	(49)
Cy5-AsNPs	Radiotherapy	AgNPs	^1^O_2_	C6 orthotopic glioma-bearing mice	Na	(50)
Au@Cu_2-x_Se NPs	Radiotherapy	Au@Cu_2-x_Se	•OH	U87 orthotopic glioma-bearing mice	Na	(51)

### 3.2 Photothermal therapy (PTT)

The principle of PTT is that photothermal agents (PTAs) convert light into heat energy when irradiated by near-infrared light to increase the temperature of the surrounding environment, thereby killing the tumor cells (59, 60). The advantages of PTT include the energy of the external light source, high efficiency and non-invasiveness (61). High enrichment in tumor tissue is one of the necessary conditions for PTAs to produce great PTT effects (62, 63).

At present, PTAs are mainly divided into two categories: inorganic materials and organic materials. Among them, inorganic materials include noble metal materials (64, 65), transition metal materials (66), carbon-based nanomaterials (such as carbon nanotubes and graphene) (67), and other two-dimensional materials (such as black phosphorus nanosheets, boron nitride, and graphitic carbon nitride) (68, 69); organic materials include near-infrared responsive small molecules and semiconducting polymers, etc (70, 71). Inorganic PTAs have higher photothermal conversion efficiency and better photothermal stability, while organic PTAs have better biocompatibility and biodegradability.

The nanomedicine-mediated PTT has been widely applied in glioma treatment. For example, Jia et al. fabricated indocyanine green (ICG)-loaded liposomes and further embedded the glioma cell membrane protein into the liposomes (BLIPO-ICG nanoparticles). With the glioma cell membrane protein, the BLIPO-ICG nanoparticles could cross the BBB and target the orthotopic glioma cells. The ICG in BLIPO-ICG nanoparticles could be used for *in vivo* fluorescence imaging. Besides, the photothermal effect of BLIPO-ICG nanoparticles could rapidly heat up under the laser irradiation, which could significantly suppress the glioma growth with an inhibition rate of 94.2%. these results. Overall, the PTT has shown great application prospect in glioma treatment.

### 3.3 Chemodynamic therapy (CDT)

CDT uses metal ion-based Fenton or Fenton-like reactions to convert less reactive H_2_O_2_ into more toxic ·OH, killing tumor cells (44, 72–75). Compared with normal cells, tumor cells can produce higher levels of H_2_O_2_. Utilizing the over-generated H_2_O_2_ in tumor as an endogenous prodrug, CDT enables tumor specific ·OH production to induce intracellular oxidative stress imbalance and reduce damage to normal tissues (76, 77). Moreover, several types of metal ions have exhibited Fenton-like activity, such as Mn^2+^, Cu^2+^, Co^2+^, Pt^2+^, *etc* (74, 78–81). However, the efficiency of CDT in tumor cells might be limited by various factors, such as limited H_2_O_2_ content in tumor cells, high level of intratumoral GSH that is able to neutralize the produced ·OH, intracellular pH and temperature, *etc* (72, 73, 82). Researchers have designed various nanoparticles to overcome these obstacles.

According to the Warburg effect, cancer cells are more dependent on glucose for nutrition than normal cells, and the glucose level in tumor cells is significantly higher than that in normal cells (83). To improve the H_2_O_2_ level in tumor cells, several studies added glucose oxidase (GOD) into the nanomedicines for enhancement of CDT efficiency (84–87). GOD can catalyze the oxidation of intratumoral glucose and generate H_2_O_2_ (88). The supplementary H_2_O_2_ can serve as substrate for the Fenton reaction, augmenting the CDT effect. In addition, hyperthermia in tumor site via PTT could also enhance CDT efficiency (89). Compared with near infrared I (NIR-I), near infrared II (NIR-II) lasers have deeper tissue penetration and higher maximum permissible exposure (MPE) (90, 91). Usually, the MPE of 808 nm (NIR-I) laser is 0.33 W/cm^2^, but the MPE of 1064 nm (NIR-II) laser is 1W/cm^2^. Human skin will burn when exposed to lasers with power higher than MPE (92). Therefore, nanomaterials with higher NIR-II absorption were chosen for PTT-enhanced CDT. Besides, GSH plays an important role in protecting cells against external damage (93). However, high levels of GSH in tumor cells can neutralize high reactive ROS, such as ·OH, thereby weakening the effect of CDT (94). Therefore, reducing the level of GSH in tumor cells can improve CDT efficiency.

**Figure 2:**
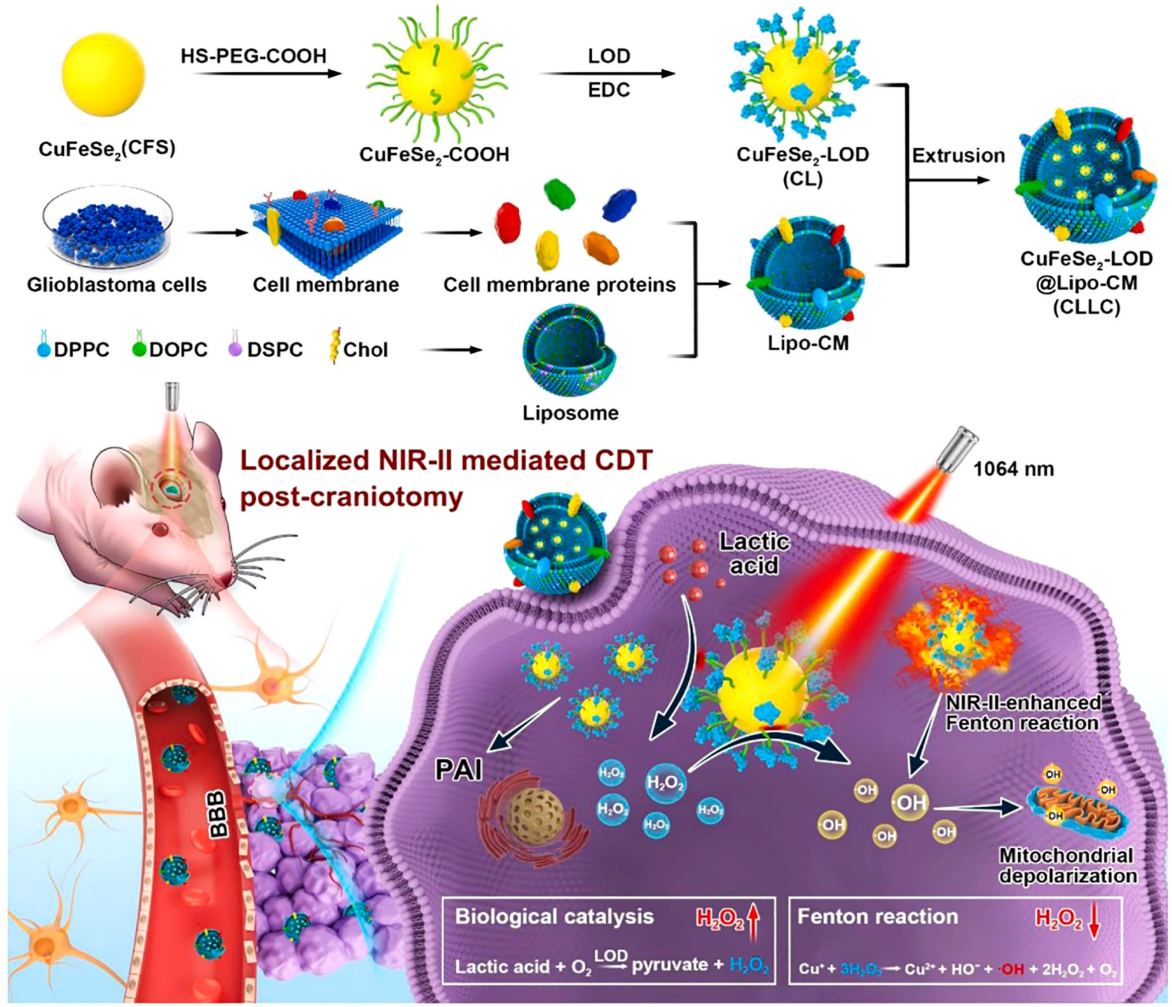
Schematic illustration of CLLC-mediated CDT of glioma. Reproduced with permission from ref (44). Copyright 2022, Elsevier.

In addition, Pan et al. proposed a localized NIR-II laser mediated CDT strategy for treatment of glioblastoma (44). They first prepared ultrasmall CuFeSe_2_ nanocrystals as CDT agent. Then, the CuFeSe_2_ nanocrystals were conjugated with lactate oxidase (LOD) and further coated with biomimetic fused liposome membrane (CuFeSe2-LOD@Lipo-CM, CLLC, **Figure 2**). The *in vivo* fluorescence imaging showed that CLLC nanocatalysts could penetrate the BBB and actively target orthotopic glioblastoma. After entering the glioblastoma cells, the Fenton-like reaction mediated by Cu^+^ in CLLC nanocatalysts could convert H_2_O_2_ into ·OH for CDT (**Figure 2**). The level of lactic acid in tumor is significantly higher than that in normal tissues. The LOD in CLLC nanocatalysts was able to catalyze oxidation of lactic acid to H_2_O_2_, which could be utilized to augment localized Fenton-like reaction. Furthermore, the photothermal conversion rate of CLLC nanocatalysts was 49.7%, indicating that the NIR-II laser mediated PTT could remarkably increase *in situ* temperature of tumor site and further enhance CDT. In addition, the mouse skulls severely block laser from entering the brain. Thus, the skull above orthotopic glioblastoma was resected to allow the laser to reach the glioblastoma site. Besides, the photoacoustic (PA) imaging could guide NIR II laser irradiation and monitor the CLLC-based CDT process. The *in vivo* therapeutic evaluation revealed that CLLC+NIR-II laser could significantly inhibit glioblastoma growth and prolong the median survival time (43 d). In addition, biological safety evaluation revealed that laser irradiation showed negligible neural toxicity on healthy mice. These results suggested that the CLLC-mediated localized CDT could be a promising treatment for glioblastoma.

Besides, Tan et al. utilized Mn^2+^-mediated Fenton-like reaction for CDT of glioma (95). They loaded temozolomide (TMZ) and manganese oxide (MnO) into an iRGD-modified polymeric micelle (iRPPA@TMZ/MnO). Due to the modification of iRGD, the iRPPA@TMZ/MnO micelles could efficiently cross the BBB and further precisely target the glioma tissues. The MnO in iRPPA@TMZ/MnO micelles could responsively degrade into Mn^2+^ and oxygen after reaching the tumor site. Simultaneously, the TMZ was rapidly released for chemotherapy of glioma cells. The Mn^2+^-mediated Fenton-like reaction could produce a large number of ·OH, inducing intracellular oxidative stress and kill the glioma cells. In addition, the generated oxygen could alleviate hypoxic tumor microenvironment, which could enhance the therapeutic efficiency of CDT and chemotherapy. Furthermore, the Mn^2+^ could also be used for T1-weighted magnetic resonance imaging (MRI) that could monitor the treatment process. *In vivo* therapeutic effect evaluation revealed that the median survival time of mice with orthotopic gliomas in iRPPA@TMZ/MnO group was 35 days, significantly longer than that in PBS group (15 days). These results suggested that iRPPA@TMZ/MnO-mediated CDT/chemotherapy could be an alternative strategy for glioma treatment.

### 3.4 Sonodynamic therapy (SDT)

Ultrasound (US), which uses mechanical vibration waves (frequency > 16 kHz) with a deep tissue penetration depth to treat and diagnose diseases, has been widely applied in clinical applications, including US imaging and high-intensity focused US (HIFU) (96). In 2004, Rosenthal et al. first define the SDT as a treatment method consisting of the synergistic effect of US and a chemical compound (sonosensitizer) (97). SDT is an emerging therapeutic modality that utilized low-intensity US to stimulate the sonosensitizers within specific tissues for generation of ROS and cavitation bubbles, thereby eliminate tumor tissues (98, 99). Due to low invasiveness and strong tissue penetration ability, SDT has gradually used to treat glioma. SDT was first used to treat glioma in 2008 (100). Until now, the mechanism of SDT for cancer therapy has not been totally uncovered. At present, it is generally believed that there are mainly two mechanisms for SDT: ROS production and ultrasonic cavitation effect (101, 102). Traditional sonosensitizers, including porphyrin and hematoporphyrin, were utilized to treat the glioma under US irradiation. However, the therapeutic efficiency was unsatisfactory, since these traditional sonosensitizers could not effectively across the BBB and accumulate in tumor regions. Moreover, the hypoxic tumor microenvironment significantly limits the SDT efficiency. Notably, with the rapid development of nanotechnology, several nanosonosensitizers were constructed to improve the antitumor efficacy of SDT (103–110).

**Figure 3:**
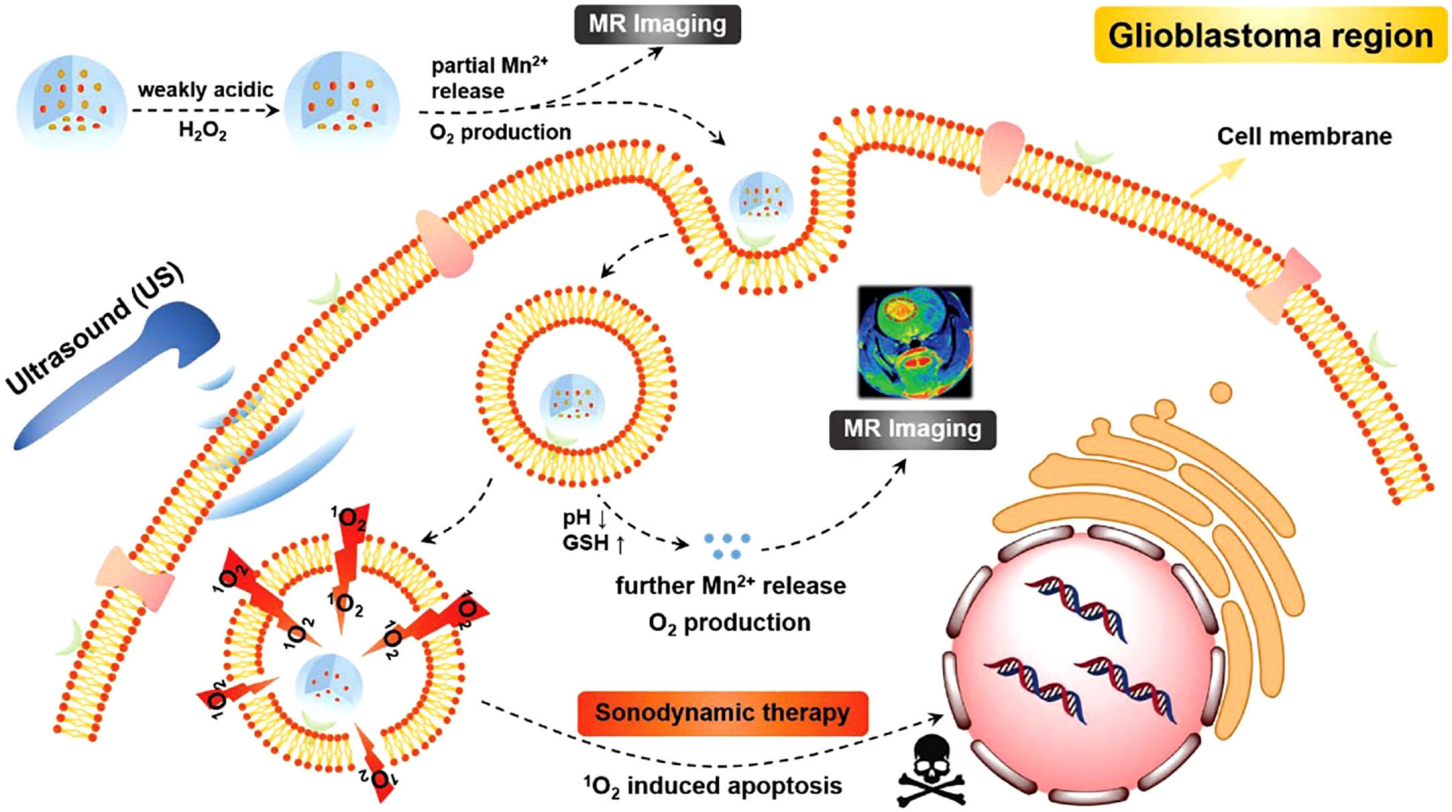
Schematic illustration of application of TMP NPs for SDT of glioblastoma. Reproduced with permission from ref (47). Copyright 2020, Wiley.

Wang group constructed an intelligent nanosonosensitizer for efficient SDT of glioblastoma (47). First, the MnO_2_ nanocrystals were grown onto the holo-transferrin (holo-Tf) via reformative biomineralization. Then, the protoporphyrin (ppIX) as a sonosensitizer was conjugated with the holo-Tf to prepare the MnO_2_@Tf-ppIX nanocomposites (TMP). The TEM results displayed that TMP nanocomposites showed uniform morphology. The zeta potential of TMP nanocomposites was -20 ± 0.2 mV, which was suitable to blood circulation and BBB crossing. Due to transferrin receptors of the BBB, the TMP nanocomposites could efficiently cross the BBB and enrich in the glioblastoma sites. Furthermore, the MnO_2_ could responsively degrade to Mn^2+^ and oxygen under the conditions of glutathione (GSH) and acid tumor microenvironment (**Figure 3**). The Mn^2^ could be utilized for T1-weighted magnetic resonance imaging (MRI) which could guide the SDT of tumor. Besides, the generated oxygen could alleviate hypoxic tumor microenvironment, which could enhance the SDT efficiency. In addition, the intratumoral TMP nanocomposites could produce a large number of ^1^O_2_, thereby induced apoptosis of tumor cells. Antitumor evaluation results showed that the tumors in tumor-bearing mice in TMP + US group (1.0 MHz, 1.5 W/cm^2^, 3 min) were fully eliminated, however, the tumor inhibition rates in other groups were unsatisfactory. Overall, the TMP-mediated SDT could be an alternative treatment approach for glioblastoma.

Indocyanine green (ICG) as a sonosensitizer has been approved by the US Food and Drug Administration (FDA) for clinical applications. Liu group loaded catalase (CAT) and ICG into silica nanoparticles (defined as CSI) for hypoxia alleviation and SDT of glioblastoma (49). Besides, the CSI nanoparticles were coated with AS1411 aptamer-conjugated macrophage exosomes (CSI@Ex-A) for enhancing BBB crossing ability. The CSI@Ex-A nanoparticles could effectively cross the BBB and target the glioblastoma cells. After entering the glioblastoma cells, the CSI@Ex-A nanoparticles could be degraded responsively in the presence of GSH, resulting in release of CAT and ICG. The CAT could catalyze (H_2_O_2_) to generate oxygen, thereby alleviating hypoxic tumor microenvironment. The ICG could produce amount of ROS under US irradiation and induce tumor cell death. In addition, both GSH resuming and supplementary oxygen could augment the SDT efficiency. The combined treatment (CSI@Ex-A + US irradiation) could remarkably suppress tumor growth and prolong the median survival time of orthotopic glioblastoma-bearing mice to 35 days, much longer than 23 days of PBS group. In addition, the CSI@Ex-A nanoparticles showed quite low toxicity to normal tissues and organs. These results suggested that the CSI@Ex-A nanoagents could be a promising sonosensitizer for glioblastoma treatment. US-targeted microbubble destruction (UTMD) is a US technique that can reversibly open the BBB and facilitate drug accumulation in glioma sites. Qu et al. fabricated an all-in-one nanosonosensitizer to enhance SDT of glioma (48). They loaded sonosensitizers chlorin e6 (Ce6) and hydroxychloroquine (HCQ, an autophagy inhibitor) into liposomes, which were further modified with targeting peptide angiopep-2 (defined as ACHL). UTMD-based BBB opening allowed ACHL to accumulate in glioma sites. The Ce6 could generate ROS under US irradiation and further induce apoptosis and MAPK/p38-PINK1-PRKN-dependent mitophagy. Besides, the released HCQ was able to inhibit autophagosome degradation, thereby amplifying intratumoral ROS generation. *In vivo* treatment evaluation revealed that ACHL-SDT could significantly suppress tumor growth and prolong median survival time of tumor-bearing mice. Therefore, the ACHL-mediated SDT might achieve precision treatment for glioma by integrating SDT-induced apoptosis and mitophagy inhibition. Overall, the SDT could be an emerging ROS-generation treatment approach for glioma.

In addition, temozolomide, the most commonly used chemotherapeutic drug of glioma could produce ROS under US irradiation and induce necroptosis in glioma, indicating that temozolomide plus US could be a promising treatment for glioma (111).

### 3.5 Radiation therapy

Radiation therapy (radiotherapy) as a crucial treatment modality which is applied to more than half of all cancer patients (112, 113). Furthermore, conventional treatment for glioma includes surgery, radiotherapy and chemotherapy. Almost most glioma patients will receive radiotherapy. In general, radiotherapy works through ionizing radiation, which includes photon radiation, including X-rays and gamma rays, but also particle radiation, such as α particles, carbon ions, β particles, electrons, neutrons, *etc* (114–116). Radiotherapy kills tumor cells through direct and indirect effects. Direct action is biomolecular damage, particularly DNA double strand breaks, leading to necrosis or apoptosis. The indirect effect refers to the promotion of apoptosis by ROS generated by the radiolysis of water molecules (116–121). Rapid development of nanotechnology remarkably improves the radiotherapy efficiency via alleviation of hypoxic tumor microenvironment, X-ray absorbance of high-Z elements, increase of tumor accumulation of radiosensitizers, *etc* (122). In addition, the nanotechnology-mediated radiosensitization has been widely utilized to treat glioma and has achieved excellent outcomes (123–125).

For example, Zhao et al. conjugated the silver nanoparticles (AgNPs) with polyethylene glycol (PEG) and As1411 to fabricate AsNPs for radiosensitization of glioma (50). *In vitro* experiments revealed that the AsNPs could actively target glioma cells and penetrate deeply into tumor spheroids. *In vivo* fluorescence imaging showed that the Cy5-AsNPs were able to effectively accumulate at the glioma region, and the fluorescence intensity of Cy5-AsNPs in glioma sites was much higher than that of PEGylated AgNPs. *In vivo* treatment efficacy evaluation revealed that the tumor inhibition rate of AsNPs + X-ray irradiation was highest and that the median survival time of glioma-bearing mice in this group was significantly extended. In addition, the AGuIX nanoparticles has been utilized in clinical trials for radiosensitization of brain metastases (NCT02820454, NCT04094077, NCT04899908, and NCT03818386). Verry et al. utilized gadolinium-based AGuIX nanoparticles for MRI-guided radiotherapy of glioma (126). The *in vivo* MRI results showed that the tumor regions could showed T1-weighted enhancement even 1 day after intravenous injection of AGuIX nanoparticles. Furthermore, the tumor growth was significantly inhibited when the tumor-bearing mice received X-ray irradiation (6 mV). Moreover, the pharmacokinetics and toxicology evaluations revealed that the AGuIX nanoparticles exhibited excellent biosafety. These results indicated that the AGuIX nanoparticles were a promising type of nanoradiosensitizers for glioma treatment.

**Figure 4:**
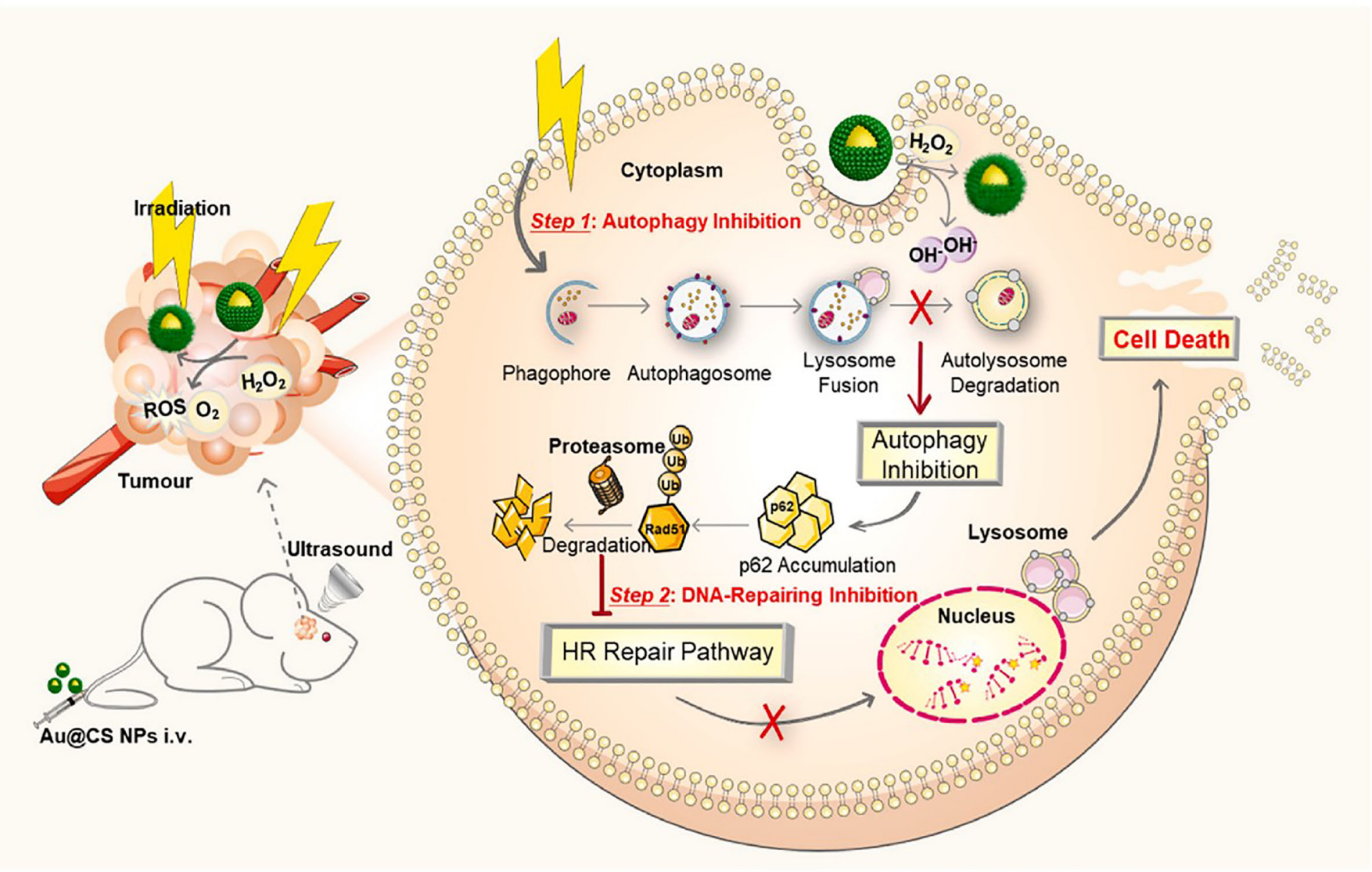
Schematic illustration of application of Au@Cu_2-x_Se NPs for glioblastoma radiotherapy. Reproduced with permission from ref (51). Copyright 2022, Elsevier.

The radioresistance leads to poor radiotherapy efficacy of glioblastoma. The resistance of tumor cells is mainly associated with their ability of damaged DNA repairing. Besides, the protective autophagy was reported to play a key role in clearance and degradation of damage organelles and DNA, thereby rescuing the damaged tumor cells (127). Thus, effective inhibition of the damaged DNA repair and protective autophagy was important for improvement of radiotherapy efficacy. Xu et al. coated gold nanoparticles with core-shell copper selenide to construct Au@Cu_2-x_Se NPs for enhancing radiotherapy of glioblastoma (**Figure 4**) (51). The prepared Au@Cu_2-x_Se NPs could alkalize lysosomes and further suppress the autophagy flux. In addition, they also found that Au@Cu_2-x_Se NPs could enhance the degradation of DNA repair protein Rad51 and decrease the DNA repair. Both suppression of DNA repair and protective autophagy could remarkably kill the glioblastoma cells via combination of Au@Cu_2-x_Se NPs and radiation. This work suggested that suppression of protective autophagy flux and DNA repair of cancer cells via well-designed nanoradiosensitizers is a promising strategy for improvement of radiotherapy efficacy of glioblastoma.

### 3.6 Synergistic therapy

Combination of different ROS-based nanomedicine strategies has been shown great synergistic therapeutic effects on glioma. For example, Zhang group prepared a nanocomposite with upconversion nanoparticle, NH_2_-MIL-53 (Fe) and DOX for PDT/CDT/chemotherapy of glioma (45). The prepared nanocomposites were further modified with lactoferrin, allowing them to cross the BBB and target the tumor cells. The upconversion nanoparticles were able to transfer the 808 nm laser into UV light to irradiate NH_2_-MIL-53 for PDT. Besides, the Fe ions in the nanocomposites could catalyze the H_2_O_2_ into ·OH for CDT. In addition, the DOX could be released under the acid tumor microenvironment for chemotherapy of glioma. The *in vivo* therapeutic evaluation showed that the synergistic CDT/PDT/chemotherapy could significantly inhibit the growth of glioma. Thus, the ROS-based multimodal therapies could be a promising treatment for glioma.

## 4 Summary and outlook

ROS-based nanomedicine provides an alternative strategy for the treatment of glioma. Nanosensitizers could catalyze the production of ROS under irradiation of light, radiation, or US as well as intracellular H_2_O_2_. In this review, we summarize the recent advances of ROS-based nanomedicine in their BBB crossing ability and application against glioma. Although promising, there are still several limitations need to be solved in future research.

Emerging BBB-crossing nanotechnology provides great opportunities to deliver chemotherapeutic drugs and sensitizers to glioma for effective treatments. These nanosensitizers could cross the BBB through AMT, CMT, RMT, *etc.* However, there are still several challenges need to be overcome to translate these nanosensitizers from bench to bedsides. The nanoparticles can absorb proteins or small molecules onto their surface after intravenous administration, leading to formation of “protein corona”. The “protein corona” might alter the surface chemistry of the nanoparticles, weakening their BBB-crossing ability and the therapeutic efficacy. Besides, although the nanosensitizers could cross the BBB and produce ROS in tumor sites, the drug concentration in the glioma is still limited. Thus, other injection methods might be considered to treat the glioma, such as intracerebral injection, intranasal administration and intrathecal injection.

The hypoxic tumor microenvironment was a major limitation for PDT. In recent years, type I PDT has been gradually reported to treat tumors, especially hypoxic solid tumor, due to its oxygen independence property (128). Therefore, the type I PDT might provide a promising treatment strategy for glioma in future. CDT is driven by endogenous factors, such as H_2_O_2_. On the one hand, the efficacy of CDT is still limited by H_2_O_2_ content in tumor cells. Thus, recent studies proposed various H_2_O_2_-supplementary strategy, such as GOD, LOD, *etc* (44, 129, 130). The GOD or LOD is able to catalyze oxidation of glucose or lactic acid to produce H_2_O_2_, thereby provide reaction material to CDT. On the other hand, the catalytic efficiency can be further enhanced by increasing intratumoral temperature and application of single atom nanozymes. SDT as a novel treatment approach has shown excellent therapy efficiency and biosafety. However, there are still some obstacles that need to be further resolved in the future. First, the potential mechanisms of antitumor effect of SDT still need to be explored in future research. Second, traditional sonosensitizers are mainly small organic molecules. There are some shortcomings to these sonosentizers, including low stability, poor BBB crossing ability, poor tumor selectivity, *etc.* Thus, future studies might focus on development of inorganic sonosentizers, such as Ti-based sonosentizers, black phosphorus, *etc.* Third, the optimal parameters of frequency and power of US for SDT need to be further studied.

Besides, the mouse glioma models are quite important for efficacy evaluation of ROS-based nanomedicine. First, the glioma models in most studies were built with homogenous glioma cell lines, which is difficult to stimulate the tumor heterogeneity in clinical patients. Second, several studies used the subcutaneous glioma models which cannot be used to assess the ability to cross the BBB. Third, most glioma models were established into immunodeficiency animals, which making it difficult to evaluate the systemic immunity induced by ROS-based nanomedicine. Thus, more suitable glioma model for ROS therapy evaluation should be developed in future research.

## Author contributions

DW conceived the study and drafted the manuscript. XC, SZ, and BL revised the manuscript critically for important intellectual content. All authors approved the final manuscript. All authors contributed to the article and approved the submitted version.
